# Spastische Parese der Arme und schlaffe Plegie der Beine bei sekundär-progredienter Multipler Sklerose

**DOI:** 10.1007/s00115-021-01103-2

**Published:** 2021-03-12

**Authors:** C. Jacksch, S. Paschen, S. Peters, V. Lindner, D. Berg

**Affiliations:** 1 Klinik für Neurologie, Universitätsklinikum Schleswig-Holstein, Campus Kiel, Arnold-Heller-Str. 3, 24105 Kiel, Deutschland; 2 Klinik für Radiologie und Neuroradiologie, Universitätsklinikum Schleswig-Holstein, Campus Kiel, Kiel, Deutschland

## Fallbericht

Wir berichten über den Fall einer 56-jährigen Patientin, die sich auf Anraten des Hausarztes zur erneuten stationären Abklärung einer 1997 erstdiagnostizierten und schwerbehindernden sekundär-progredienten Encephalomyelitis disseminata vorstellte. Es bestand ein hoher Leidensdruck aufgrund einer mittlerweile vollständigen Immobilität. Anamnestisch war ein Beginn mit okulären Symptomen, retrospektiv am ehesten einer Retrobulbärneuritis entsprechend, zu eruieren. Im Verlauf sei es zu einer progredienten Störung des Gangbildes gekommen. Die Patientin hatte über die Jahre zwar intermittierende Kortisonstoßtherapien erhalten, jedoch nie eine schubprophylaktische Therapie.

Weiterhin bestand verkomplizierend ein langjähriger Opiat- und Benzodiazepinabusus. In der klinisch-neurologischen Untersuchung bei Aufnahme bot sich ein für uns zunächst überraschender klinischer Befund aus proximal und linksbetonter Armparese (proximal KG [Kraftgrad] 3/5, distal 4/5), linksseitig mit mittelgradiger spastischer Komponente sowie ein an der unteren Extremität hierzu paradoxer Befund mit einer hochgradigen schlaffen Paraparese (proximal KG 1/5, distal 2/5). Der Reflexstatus an der oberen Extremität zeigte sich lebhaft mit teils verbreiterten Reflexzonen, während die Muskeleigenreflexe der unteren Extremität ubiquitär erloschen waren bei beidseits positivem Zeichen nach Babinski. Des Weiteren gab die Patientin eine diffuse Hypästhesie des gesamten Körpers an und klagte über eine Dranginkontinenz bei erhaltener Kontrolle über die Defäkation.

Aufgrund des diskrepanten klinischen Befundes begannen wir bei anamnestisch nicht rekonstruierbarer Vordiagnostik eine erneute umfangreiche Abklärung.

Laborchemisch fand sich ein leichter Folsäure- und ein deutlicher Vitamin-D-Mangel. Der Wert für Vitamin B12 zeigte sich normwertig, weiterhin fanden sich in der erweiterten Erregerdiagnostik (inklusive Lues und Borrelien) keine positiven Befunde.

**Figure 1:**
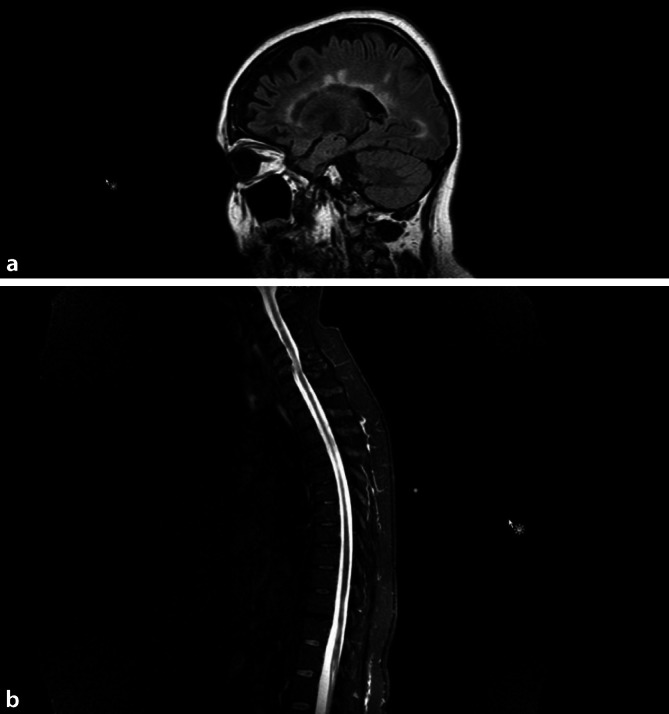

In der kranialen kontrastmittelgestützten MRT zeigte sich eine ausgeprägte Läsionslast in allen erfassten MS-typischen Lokalisationen (juxtakortikal, periventrikulär, infratentoriell) sowie eine deutlich ausgeprägte Hirnvolumenminderung (Abb. 1a). In der ergänzten MRT des Myelons zeigten sich polytope Gliosen (am Übergang der Medulla oblongata zum zervikalen Myelon, Höhe HWK 3, HWK 5/6, langstreckig auf Höhe HWK 4/5 und BWK 11/LWK 1) mit Punctum maximum im Bereich des kaudalen Myelons mit konsekutiver Atrophie (Abb. 1b). Es zeigten sich keine floriden KM-aufnehmenden Läsionen.

Zum Ausschluss einer koinzidentellen peripheren Neuropathie ergänzten wir eine umfangreiche elektrophysiologische Diagnostik, welche bei regelrechter motorischer und sensibler Neurographie und gut erhaltenen F‑Wellen keinen erhärtenden Hinweis hierfür fand. Die mit abgeleiteten MEP und SEP zeigten sich mit einer zentralen Efferenz- und Afferenzstörung vereinbar.

Der Liquor war bezüglich Zellzahl und basaler Liquorchemie unauffällig bei lediglich leichter Eiweißerhöhung (572 mg/l). Isolierte oligoklonale Banden waren nachweisbar, weiter waren 2 Komponenten der MRZ-Reaktion positiv. Der aus differenzialdiagnostischen Gründen erfolgte Test auf das Vorhandensein von Aquaporin-4- und Anti-MOG-Antikörpern verlieb negativ.

## Diskussion

Zusammenfassend erachteten wir die bestehende Diagnose einer sekundär-progredienten Encephalomyelitis disseminata unter Berücksichtigung der aktuellen McDonald-Kriterien von 2017 [1] als richtig. Das Fehlen einer akut entzündlichen Läsion sowie die ausgeprägte Atrophie an Hirnsubstanz unterstreichen einmal mehr den Wechsel von einem entzündlichen zu einem neurodegenerativen sich verselbstständigenden Charakter nach langer Krankheitsdauer [2].

Die klinisch anfänglich für uns nur schwer zu fassende Kombination aus einerseits spastischer Armparese und konträr hierzu vorliegender schlaffer Paraparese könnte letztlich nach Ausschluss konkurrierender Ätiologien (peripher entzündlich oder infektiöse Neuropathie) das Ergebnis der ausgeprägten Gliose und Atrophie mit Schwerpunkt im Bereich des kaudalen Myelons proximal des Konus mit hierdurch bedingtem multisegmentalem Ausfall der spinalen Stellreflexe sein. Eine Übersicht über Differenzialdiagnosen dieses klinischen Bildes bietet Tab. 1.

**Table 1:** 

	Beispielerkrankungen	Zusatzdiagnostik
Vaskulär	Spinale Ischämie Spinale Blutung Gefäßmalformationen	sMRT mit MR-Angiographie DSA
Strukturell	Spinale Tumoren Syringomyelie	sMRT mit Kontrastmittel Liquordiagnostik
Metabolisch	Funikukläre Myelose	Anamnese Labordiagnostik: Vitamin B12, Holo-Transcobalamin, Methylmalonsäure sMRT
Infektiös	Neuroborreliose Neurolues	Anamnese Serologie
Sonstige	Strahlenmyelopathie	Anamnese Bildgebung, EMG

Etwas diskrepant zeigen sich die elektroneurographisch noch gut erhaltenen F‑Wellen, welche auf einen intakten Regelkreis mit der antidrom stimulierten Vorderhornzelle schließen lässt. Dies lässt vermuten, dass dem ursächlichen Pathomechanismus eine aufgrund der ausgeprägten Gliose und Atrophie des kaudalen Myelons aufgehobene Kopplung zentraler auf periphere Efferenzen auf spinaler Ebene zugrunde liegt mit resultierender schlaffer Lähmung, ähnlich einer Affektion des motorischen Vorderhorns oder des peripheren Nervs als solchen. Bei gleichzeitiger Affektion der Pyramidenbahn resultieren dann, wie im vorliegenden Fall auch, positive Pyramidenbahnzeichen. Schlussendlich zeigt sich die Lösung eines zunächst klinisch irritierenden Befundes abseits apparativer Zusatzuntersuchungen in der Vergegenwärtigung der neuroanatomischen Verhältnisse.

## Fazit für die Praxis

Der Untersuchungsbefund einer schlaffen Paraparese ist auch mit ausgedehnten zerebralen und spinalen Entmarkungsherden vereinbar und muss nicht immer durch eine zusätzliche periphere Neuropathie oder Affektion des 2. Motoneurons bedingt sein.

Die Elektroneurographie und die MR-Tomographie stellen wichtige Ergänzungen zur Anamnese und zum klinischen Untersuchungsbefund in unklaren Fällen zur Differenzierung zentraler von peripherer Neuropathien dar.
